# Neutrophil-to-lymphocyte ratio and systemic inflammation response index as biomarkers for the clinical outcomes of intracerebral hemorrhagic stroke patients: a prospective cohort study

**DOI:** 10.3389/fneur.2025.1616128

**Published:** 2025-07-22

**Authors:** Ziyi Hu, Wei Zhu, Chaofeng Fan, Yan Jiang

**Affiliations:** ^1^Department of Nursing, West China Hospital/West China School of Nursing, Sichuan University, Chengdu, China; ^2^Department of Neurosurgery, West China Hospital/West China School of Nursing, Sichuan University, Chengdu, China

**Keywords:** intracerebral hemorrhagic stroke, MRS, neutrophil-to-lymphocyte ratio, systemic inflammation response index, prognosis

## Abstract

**Purpose:**

To examine the associations between the neutrophil-to-lymphocyte ratio (NLR), systemic inflammation response index (SIRI) and clinical outcomes of intracerebral hemorrhagic (ICH) stroke patients.

**Methods:**

This prospective cohort study recruited and investigated longitudinally 294 ICH stroke patients in a general tertiary hospital in Sichuan Province, China at baseline (admission), 1-month post-discharge, 3-month post-discharge and 6-month post-discharge from January 2020 to January 2022. We calculated the NLR and SIRI from blood samples collected at baseline. The Mann–Whitney test, logistic regression analysis and receiver operating characteristic (ROC) analysis were performed to evaluate differences in the NLR and SIRI between hemorrhagic stroke patients at three follow-up time points. The interaction between these variables was evaluated via multiplicative and additive interaction models.

**Results:**

Our study revealed that the cut-off values of the NLR and SIRI to predict the clinical outcomes were determined to be 6 and 4, respectively. NLR > 6 (OR 2.202, 95% CI: 1.094–4.430) and SIRI>4 (OR 2.056, 95% CI: 1.065–3.968) were associated with increased risks for poor clinical outcomes at 1-month post-discharge. SIRI>4 (OR 2.428, 95% CI: 1.389–4.243) were associated with increased risks for poor clinical outcomes at 3-month post-discharge. NLR > 6 (OR 1.978, 95% CI: 1.093–3.580) were associated with increased risks for poor clinical outcomes at 6-month post-discharge.

**Conclusion:**

The NLR and SIRI did not have an additive effect on the clinical outcome at 1-month post-discharge. Our findings indicate that high NLRs and SIRIs, particularly NLR > 6 and SIRI>4, are associated with poor clinical outcomes in ICH stroke patients.

## Introduction

Stroke is the leading cause of death in China and a significant contributor to global disability, with the second-highest rank in both mortality and disability (1, 2). Hemorrhagic stroke, comprising intracerebral hemorrhage (ICH) and subarachnoid hemorrhage (SAH), is the second most prevalent type and involves catastrophic vessel rupture, leading to brain and subarachnoid bleeding (3). Despite medical advancements, ICH stroke remains a significant public health burden (4). The variability in clinical prognosis necessitates the identification of prognostic biomarkers (3, 5). The prediction of outcomes is critical for treatment optimization and resource allocation (6). Despite considerable research, predicting outcomes in ICH stroke patients remains challenging, highlighting the urgent need for easily measurable biomarkers to guide treatment decisions and improve patient outcomes.

Inflammatory indices constitute an innovative metric that amalgamates multiple routine blood-based inflammatory markers, including the neutrophil-to-lymphocyte ratio (NLR), systemic inflammatory response index (SIRI), triglyceride-glucose index (TyG), and platelet-to-lymphocyte ratio (PLR) (5, 7, 8). These integrated biomarkers exhibit enhanced attributes of simplicity, cost-effectiveness, and stability when contrasted with individual conventional inflammatory markers. They provide a comprehensive reflection of various inflammatory and immune pathways within the organism, facilitating the assessment of systemic inflammatory burden, elucidation of innate injury mechanisms, and tracking of dynamic changes associated with ICH stroke conditions (7, 8). Intracerebral and subarachnoid hemorrhages trigger rapid blood collection in the central nervous system and elicit inflammatory immune responses involving both resident and infiltrating immune cells. Secondary injury and cerebral edema after cerebral hemorrhage are key factors leading to poor prognosis in patients with ICH stroke (9). Previous studies have suggested that the compression of surrounding tissues and microcirculation by hematoma after hemorrhage is the main cause of cerebral edema in patients with ICH stroke (10). Research on inflammatory factors suggests that inflammatory biomarker play important roles in cerebral edema after ICH stroke (11). However, most of the research on this topic is cross-sectional or retrospective (7, 8, 12, 13), and longitudinal studies examining the predictive value of biomarkers such as the NLR and SIRI over time are lacking. The trajectory of changes in the relationships between inflammatory index levels and follow-up clinical outcomes remains unclear. Given the pivotal role of inflammation in ICH stroke, understanding and monitoring biomarkers such as these are crucial for prognostic assessment (12–14). These biomarkers may reflect the intensity and direction of the inflammatory process after stroke, suggesting potential targets for immunomodulatory therapies to improve patient outcomes (3, 15).

This study aimed to investigate whether the inflammatory biomarker NLR and SIRI are independently associated with poor clinical outcomes in ICH stroke patients. This prospective cohort study has been reported in line with the STROCSS guidelines (Supplementary Table S1) (16).

## Methods

### Participants

Our research is a prospective cohort study. From January 2020 to January 2022, ICH stroke patients in a general tertiary hospital in Sichuan Province, China, were recruited and longitudinally investigated at baseline (admission), 1-month post-discharge, 3-month post-discharge and 6-month post-discharge. Our inclusion criteria included (1) age ≥18 years, (2) a radiologically confirmed diagnosis of intracerebral hemorrhage (ICH), and (3) complete clinical data. The exclusion criteria were as follows: (1) had combined acute or chronic infection; and (2) had combined severe heart, lung, liver, or kidney disease (Supplementary Table S2). Recruitment occurred during hospitalization via daily electronic medical record (EMR) reviews by trained research nurses. Our study was centrally approved by the Institutional Review Board (IRB: 2019–1,167) and performed in accordance with the Declaration of Helsinki. All patients provided written informed consent and all procedures were performed in accordance with relevant guidelines and regulations.

### Sample size estimation

Based on prior ICH cohort studies (7), the sample size was calculated based on the comparison of median NLR values between good and poor outcome groups. Using preliminary data showing median NLR of 4.79 (IQR: 2.82–8.95) versus 10.30 (IQR: 6.46–15.60), we estimated the standard deviation from interquartile ranges (SD ≈ IQR/1.35) yielding pooled SD = 5.76. With *α* = 0.05 (two-tailed), power = 80%, and effect size (median difference) = 5.51, the required sample size was calculated as: *n* = [2π(SD^2^)(Zα + Zβ)^2^]/(*μ*₁-μ₂)^2^ = 55 per group. Accounting for 20% attrition, we planned to enroll 69 patients per group at least (total *N* = 138). Our enrolled cohort (*n* = 294) provided adequate statistical power.

### Assessment

The study population consisted of patients who underwent comprehensive assessments, including demographic information (age, sex, ethnicity, marital status), risk factors (hypertension, diabetes status, surgical history for stroke, alcohol and smoking history, BMI), and baseline clinical characteristics (systolic/diastolic blood pressure, neutrophils, lymphocytes, and platelets). Hematology indicators, including fasting blood glucose and triglyceride levels, were measured within 24 h after admission. The neutrophil to lymphocyte ratio (NLR) were calculated as the neutrophil count divided by the lymphocyte count (7). The SIRI value was calculated as the product of neutrophil count multiplied by monocyte count divided by lymphocyte count (17). All participants underwent serial assessments using the modified Rankin Scale (mRS) at baseline and during post-discharge follow-ups (1-, 3-, and 6-month). Baseline assessments were conducted in-person during hospitalization, with standardized telephone interviews employed for outcome ascertainment at follow-ups. The mRS was used to assess functional outcomes as the primary outcome, as it is the gold standard for stroke disability evaluation (14). Patients were categorized into two groups according to these mRS scores: those with mRS scores of 0–2 indicated a better clinical outcome, and those with mRS scores of 3–6 indicated a poor clinical outcome.

### Statistical analyses

All continuous variables were assessed for normality using Kolmogorov–Smirnov tests (*α* = 0.05). For normally distributed data, the results are reported as the means±SDs. Nonnormally distributed data are presented as the median with quartiles M (Q1, Q3). The frequency was used to describe the count data. To compare categorical variables, the chi-square test was applied, independent Student’s t test was used for normally distributed variables, and the Mann–Whitney U test was used for nonnormally distributed variables. Receiver operating characteristic (ROC) curve analysis was used to identify the optimal cut-off values for the NLR and SIRI as prognostic indicators for clinical outcome. The cut-off value corresponding to the highest Youden index was selected as the threshold for distinguishing between the two groups. For the additive effects model, the delta method was used to construct the interaction term “NLR > 6 * SIRI>4.” Synergistic effect analysis between NLR > 6 and SIRI>4 was specifically conducted at the 1-month post-discharge time point. This selection was based on the prerequisite for interaction analysis that both dichotomized biomarkers demonstrated statistically significant independent associations with the primary outcome at this specific time point. Analyses at other time points were not pursued for interaction given the absence of significant main effects for at least one biomarker. Subsequently, the covariance matrix was input into the specialized calculation table in Microsoft Excel compiled by Anderson et al. (18). The point estimations and their 95% CIs for three additive interaction evaluation biomarkers were obtained: the relative excess risk of interaction (RERI), the attributable proportions of interaction (API), and the synergy index (S). Evaluation biomarkers were used to ascertain the presence of additive interactions. The absence of an additive interaction between the two factors was indicated when the point estimations and 95% CI of the RERI or the API encompassed 0 or when the same measures for the S encompassed 1. Statistical significance was determined at *p* < 0.05 (two-tailed). All the statistical analyses were performed via SPSS IBM version 27.0.

## Results

### Study population

**Figure 1:**
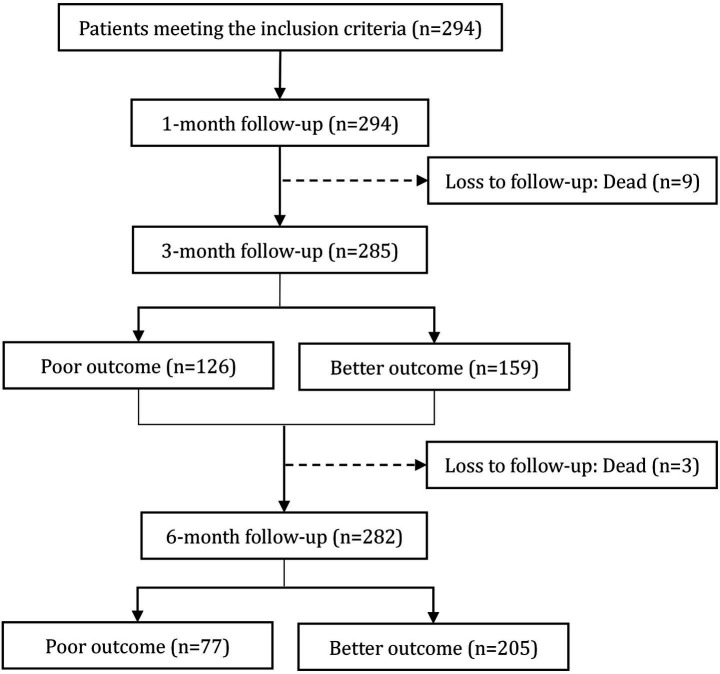
Flow chart of the study design.

The final sample included 294 patients (178 males, 60.54%) aged 55 (47, 65) years (range 18–86). Table 1 summarizes the demographic and clinical data of the participants at baseline (Neutrophil, monocyte and lymphocyte counts are listed in the Supplementary Table S3). The flow chart of the study design is as illustrated in Figure 1.

**Table 1:** Univariate analysis of factors and clinical outcomes (*n* = 294).

Characteristics	1-month post-discharge		Z/F/χ^2^	*p*	3-month post-discharge^a^		Z/F/χ^2^	*p*	6-month post-discharge^b^		Z/F/χ^2^	*p*
	Poor outcome^*^ (*n* = 135)	Better outcome^*^ (*n* = 159)			Poor outcome^*^ (*n* = 100)	Better outcome^*^ (*n* = 194)			Poor outcome^*^ (*n* = 89)	Better outcome^*^ (*n* = 205)		
Age (y), M (Q1, Q3)	56 (49, 66)	55 (46, 64)	−1.375	0.169	55.5 (49.0, 65.5)	55.00 (46.00, 65.25)	−0.835	0.404	55 (48, 66)	55 (46, 64)	−0.668	0.504
Sex, *n* (%)			1.043	0.307								
Male	86 (63.70%)	92 (57.86%)			61 (61.00%)	117 (60.31%)	0.013	0.909	53 (59.55%)	125 (60.98%)	0.053	0.818
Female	49 (36.30%)	67 (42.14%)			39 (39.00%)	77 (39.69%)			36 (40.45%)	80 (39.02%)		
Ethnic Minority			0.420	0.517			0.025	0.755			0.050	0.824
Han	131 (97.04%)	152 (95.60%)			97 (97.00%)	186 (95.88%)			86 (96.63%)	197 (96.10%)		
Other	4 (2.96%)	7 (4.40%)			3 (3.00%)	8 (4.12%)			3 (3.37%)	8 (3.90%)		
Marital Status			0.098	0.754			2.953	0.086			0.200	0.655
Married	118 (87.41%)	137 (86.16%)			82 (82.00%)	173 (89.18%)			76 (85.39%)	179 (87.32%)		
Single/Divorced/ Separation/Widow	17 (12.59%)	22 (13.84%)			18 (18.00%)	21 (10.82%)			13 (14.61%)	26 (12.68%)		
Hypertension			6.148	0.013			4.257	0.039			0.928	0.335
Yes	106 (71.52%)	104 (65.41%)			79 (79.00%)	131 (67.53%)			67 (75.28%)	14369.76 (%)		
No	29 (21.48%)	55 (34.59%)			21 (21.00%)	63 (32.47%)			22 (24.72%)	62 (30.24%)		
Diabetes			0.740	0.390			0.533	0.465			0.148	0.700
Yes	16 (11.85%)	14 (8.81%)			12 (12.00%)	18 (9.28%)			10 (11.24%)	20 (9.76%)		
No	119 (88.15%)	145 (91.19%)			88 (88.00%)	176 (90.72%)			79 (88.76%)	185 (90.24%)		
Smoking History			0.740	0.390			0.240	0.624				
Yes	16 (11.85%)	14 (8.81%)			9 (9.00%)	21 (10.82%)			9 (10.11%)	21 (10.24%)	0.001	0.973
No	119 (88.15%)	145 (91.19%)			91 (91.00%)	173 (89.18%)			80 (89.89%)	184 (89.76%)		
Alcohol History			0.026	0.872			0.866	0.352			0.266	0.606
Yes	12 (8.89%)	15 (9.43%)			7 (7.00%)	20 (10.31%)			7 (9.76%)	20 (7.87%)		
No	123 (91.11%)	144 (90.57%)			93 (93.00%)	174 (89.69%)			82 (90.24%)	185 (92.13%)		
Received Surgery for Stroke			1.177	0.278			0.002	0.969			0.177	0.674
Yes	51 (37.78%)	70 (44.03%)			41 (41.00%)	80 (41.24%)			35 (39.33%)	86 (41.95%)		
No	84 (62.22%)	89 (55.97%)			59 (59.00%)	114 (58.76%)			54 (60.67%)	119 (58.05%)		
BMI (kg/m^2^)	23.98 ± 3.47	24.44 ± 4.03	1.004	0.343	24.19 ± 3.33	24.26 ± 4.00	2.075	0.889	24.07 ± 3.65	24.31 ± 3.86	0.084	0.650
SBP (mmHg)	152.90 ± 21.88	151.82 ± 26.21	4.604	0.705	152.44 ± 22.093	152.25 ± 25.39	2.043	0.949	152.55 ± 22.93	152.21 ± 24.90	1.532	0.912
DBP (mmHg)	92.72 ± 15.99	91.12 ± 17.34	2.570	0.415	92.17 ± 16.63	91.69 ± 16.82	0.776	0.816	92.44 ± 16.88	91.60 ± 16.69	0.350	0.694
NLR	9.51 (5.57, 16.20)	6.62 (4.15, 11.85)	−3.603	<0.001	9.56 (5.51, 16.41)	7.31 (3.65, 12.67)	−2.617	0.009	9.51 (5.68, 15.87)	7.92 (4.76, 13.10)	−2.007	0.045
SIRI	5.23 (2.84, 9.26)	2.99 (1.94, 6.23)	−3.945	<0.001	5.23 (2.76, 8.90)	3.47 (1.97, 7.09)	−2.659	0.008	4.84 (2.59, 8.25)	3.63 (2.07, 7.41)	−1.694	0.090

^*^Poor outcome: mRS > 2, better outcome: mRS ≤ 2; ^a^ Dead patients (*n* = 9) were imputed as the worst possible score, counted as poor clinical outcome (mRs 3–6 dichotomy); ^b^ Dead patients (*n* = 3) was imputed as the worst possible score, counted as poor clinical outcome (mRs 3–6 dichotomy). BMI, body mass index; SBP, systolic blood pressure; DBP, diastolic blood pressure; NLR, neutrophil-lymphocyte ratio; SIRI, systemic inflammatory response index.

### Clinical outcome score and dichotomy frequency

The mRS scores at 1-month post-discharge, 3-month post-discharge and 6-month post-discharge were 2 (1, 4), 1 (1, 3), and 1 (1, 3), respectively, and the patients were divided into two groups (Table 1). The incidence of poor clinical outcomes (mRS score >2) at 1, 3, and 6-month post-discharge was 45.92, 34.01, and 30.27%, respectively.

### Univariate analysis of factors and clinical outcomes

Comparisons of combined demographic information, risk factors and clinical characteristics between the poor outcome and better outcome groups are listed in Table 1. For the clinical outcomes at 1-month post-discharge, hypertension, NLR and SIRI were significantly different between the poor outcome group and the better outcome group (*p* < 0.05). For clinical outcomes at 3-month post-discharge, hypertension, NLR and SIRI of the poor outcome group were significantly different from those of the better outcome group (*p* < 0.05). For the clinical outcome at 6-month post-discharge and NLR of the poor outcome group were significantly different from those of the better clinical outcome group (*p* < 0.05).

### ROC curves of clinical outcomes

**Figure 2:**
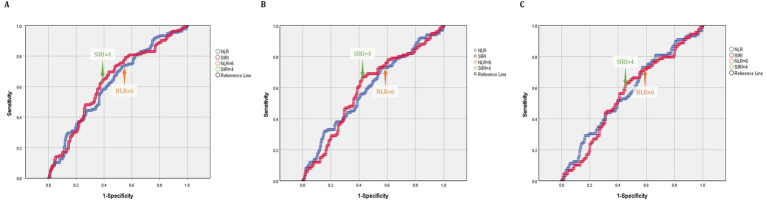
ROC curves showing the values of the NLR and SIRI for evaluating the clinical outcomes of ICH stroke patients at 1- **(A)**, 3- **(B)** and 6- **(C)** month post-discharge.

As Table 2 showed, for poor 1-month outcomes, receiver operating characteristic (ROC) curve analysis revealed that the area under the curve (AUC) values for the NLR and SIRI were 0.622 and 0.633, respectively (Figure 2A). The calculated Youden biomarker for the NLR and SIRI were 0.205 and 0.262, corresponding to the cut-off values of 6.18 and 3.59, respectively. For poor 3-month outcomes, ROC curve analysis revealed that the AUCs for the NLR and SIRI were 0.593 and 0.595 (Figure 2B). The calculated Youden biomarker for the NLR and SIRI were 0.168 and 0.247, corresponding to the cut-off values of 6.20 and 3.95. For poor 6-month outcomes, receiver operating characteristic (ROC) curve analysis revealed that the area under the curve (AUC) values for the NLR and SIRI were 0.574 and 0.562 (Figure 2C). The calculated Youden biomarker for the NLR and SIRI were 0.160 and 0.176, corresponding to the cut-off values of 6.20 and 3.95. By integrating the cutoff values of the three follow-up time points, the cutoff values of the NLR and SIRI were determined to be 6 and 4 (Table 3).

**Table 2:** Youde index, cut-off values and AUCs of follow-up clinical outcomes.

Clinical outcome		NLR	SIRI
1-month post-discharge	Cut-off value	6	4
	Youden index	0.205	0.262
	AUC	0.622	0.633
3-month post-discharge	Cut-off value	6	4
	Youden index	0.168	0.247
	AUC	0.593	0.595
6-month post-discharge	Cut-off value	6	4
	Youden index	0.160	0.176
	AUC	0.574	0.562

**Table 3:** Frequency analysis of NLR and SIRI using cut-off values for classification (*n* = 294).

Variable		*n* (%)
NLR	≤6	200 (68.03%)
	>6	94 (31.97%)
SIRI	≤4	147 (50.00%)
	>4	147 (50.00%)

### Binary logistic regression analysis and synergistic effect analysis

By using the cut-off values of the NLR and SIRI, the patients were divided into two groups, and the frequencies are shown in Table 4. Binary logistic regression analysis was conducted. The independent variables were age (actual value), sex (coded: male = 1, female = 0), ethnic minority status (coded: han = 1, other = 0), marital status (coded: married = 1, single/divorced/separation/widow = 0), hypertension (coded: yes = 1, no = 0), diabetes (coded: yes = 1, no = 0), smoking history (coded: yes = 1, no = 0), alcohol history (coded: yes = 1, no = 0), BMI (actual value), SBP and DBP at admission (actual value), NLR (>6 = 1, ≤6 = 0) and SIRI (>4 = 1, ≤4 = 0), with the dependent variable being the clinical outcome of the patients (coded: poor = 1, better = 0). The findings revealed that at 1-month post-discharge, age (OR = 1.032, *p* = 0.004), NLR > 6 (OR = 2.202, *p* = 0.027) and SIRI>4 (OR = 2.056, *p* = 0.032) were significant predictors of clinical outcome. At 3-month, SIRI>4 (OR = 2.428, *p* < 0.001) remained influential. At 6-month post-discharge, the clinical outcome was significantly affected by NLR > 6 (OR = 1.978, *p* = 0.024) (Table 5).

**Table 4:** Index of additive interaction between NLR and SIRI on 1-month post-discharge poor clinical outcome (*n* = 294).

Evaluation biomarkers	Model 1	Model 2
RERI	0.901 (−3.120, 4.922)	2.027 (−4.520, 8.575)
API	0.289 (−0.652, 1.231)	0.394 (−0.398, 1.185)
S	1.744 (0.237, 12.835)	1.954 (0.379, 10.082)

Model 1: not adjusted; Model 2: adjusted for age, sex, ethnic minority status, marital status, surgery for stroke, hypertension, diabetes status, smoking history, alcohol history, BMI, SBP and DBP at admission. RERI, relative excess risk of interaction; API, attributable proportions of interaction; S, synergy index.

**Table 5:** Binary logistic regression analysis of NLR and SIRI using cut-off values for classification (*n* = 294).

Variable		OR (95% CI)	*p*
1-month post-discharge	Age	1.032 (1.010–1.055)	0.004
	NLR > 6	2.202 (1.094–4.430)	0.027
	SIRI>4	2.056 (1.065–3.968)	0.032
3-month post-discharge	SIRI>4	2.428 (1.389–4.243)	<0.001
6-month post-discharge	NLR > 6	1.978 (1.093–3.580)	0.024

To analyze the synergistic effect of the NLR and SIRI at 1-month post-discharge, the biomarkers were multiplied pairwise to generate a new variable. After with or without controlling for potential confounders, the additive interactions between the NLR and the SIRI were analyzed via binary logistic regression. The multiplicative interactions between the NLR and the SIRI did not significantly affect patients’ 1-month clinical outcome (*p* > 0.05) (Tables 4, 6).

**Table 6:** Multiplicative interactive effect of NLR and SIRI on poor clinical outcomes at 1-month post-discharge (*n* = 294).

Variable		1-month post-discharge		
		*β*	OR (95% CI)	*p*
Model 1	NLR	0.30319	1.354 (0.668–2.745)	0.400
	SIRI	0.61904	1.857 (0.575–6.002)	0.301
	NLR*SIRI	0.21308	1.237 (0.323–4.746)	0.756
Model 2	NLR	0.77523	2.171 (0.920–5.126)	0.077
	SIRI	0.66971	1.954 (0.485–7.868)	0.346
	NLR*SIRI	0.19449	1.215 (0.247–5.968)	0.811

Model 1: not adjusted; Model 2: adjusted for age, sex, ethnic minority status, marital status, surgery for stroke, hypertension, diabetes status, smoking history, alcohol history, BMI, SBP and DBP at admission. NLR, neutrophil-lymphocyte ratio; SIRI, systemic inflammatory response index; BMI, body mass index; SBP, systolic blood pressure; DBP, diastolic blood pressure.

## Discussion

Consistent with previous reports on inflammatory biomarkers in stroke, we observed that elevated NLR (>6) and SIRI (>4) were independently associated with prognosis of the ICH patients. Inflammation plays a pivotal role in the pathogenesis of stroke (3, 19), with key mediators —specifically IL-1β (neutrophil recruitment), IL-6 (acute-phase amplification), and TNF-*α* (neuronal apoptosis)— confirmed to drive secondary injury and correlate with ICH severity/prognosis (19, 20). Compared with the original indicators, the NLR and SIRI, which are emerging inflammatory biomarker, have the advantage of being more sensitive (21, 22), but there has not yet been a consistent conclusion on the prediction of outcomes in patients with ICH stroke. The NLR and SIRI are attractive systemic inflammatory biomarkers because they are inexpensive, simple, routine, rapid, reproducible markers of inflammation and are easily obtained from a routine complete blood cell count with differential measurements (23, 24).

Despite growing interest in the predictive value of the NLR and SIRI at hospital admission, the literature presents a complex picture with varying results and inconsistent conclusions regarding their prognostic utility (7, 12). To the best of our knowledge, longitudinal follow-up studies on inflammatory biomarker in ICH stroke patients are rare. This study aims to clarify the role of these biomarkers in the longitudinal prognosis of ICH stroke patients, thereby addressing the current gaps in knowledge and contributing to the ongoing debate on their clinical application. Our findings align with previous studies that reported a correlation between these inflammatory markers and poor clinical outcomes, with the incidence rates of poor outcomes (mRS score >2) at 1-, 3-, and 6- month post-discharge being 45.92, 34.01, and 30.27%, respectively. The incidence rates were similar to those reported in previous studies (25, 26). The observed consistency in incidence rates across studies may stem from the fundamental biological mechanisms linking systemic inflammation (as reflected by NLR/SIRI) to neuronal damage and impaired recovery, which operate similarly across populations. Patients in the two groups with different clinical outcomes demonstrated significant differences in characteristics, including hypertension, NLR and SIRI. Most characteristics exhibit stronger differences in the early stages. These indicators reflected the severity of the condition, the inflammatory status, and the immune response, which collectively influence the prognosis of patients (7). The time-dependent nature of post-stroke inflammation, where early-phase inflammatory cascades (1-month) have more pronounced effects on tissue damage than later chronic inflammation (6-month). The stronger early-stage differences in hypertension and inflammatory markers suggest their role as acute-phase drivers of secondary injury, while the declining incidence rates over time may reflect either natural resolution of inflammation or survivor bias in longitudinal follow-up.

To delve deeper into the clinical utility of these inflammatory biomarker, we performed an ROC analysis to determine the optimal cut-off values for the NLR and SIRI. After dichotomizing the NLR and SIRI on the basis of the determined cut-off values, we conducted binary logistic regression analysis. The results revealed that age, hemorrhage location, the NLR (>6) and the SIRI (>4) exhibited varying predictive capabilities for clinical outcomes at different follow-up time points post-discharge. Notably, at the 1-month follow-up, both the NLR (>6) and the SIRI (>4) emerged as independent predictors of clinical outcome, a finding that was similar to that of the study by Li (27), suggesting the validity of these cut-off values. While NLR/SIRI show limited long-term predictive power, their 1-month prognostic capacity supports utility as simple triage tools. Integration with clinical/imaging variables may enhance multi-phase prediction (26). The synergistic effect analysis results indicated no significant interactions between NLR and SIRI on 1-month post-discharge clinical outcomes (S = 1.954) (18). Further analysis of the interaction between the NLR (>6) and the SIRI (>4) indicated the absence of a synergistic effect. Consequently, clinicians may consider NLR (>6) and SIRI (>4) as valuable references in the prognostic assessment of patients with ICH stroke.

The above findings can be summarized in two points. First, these observations suggest that the NLR and SIRI are potential biomarkers for predicting the clinical outcome of ICH stroke patients because of the key role of lymphocytes in immune regulation, which is similar to the conclusions of previous studies (26, 28, 29). The newly generated variables NLR > 6 and SIRI>4, following dichotomization by cut-off values, exhibited even greater predictive power for the clinical outcomes of patients. Critically, their divergent predictive power across timepoints stems from inherent biological differences: SIRI incorporates monocytes—key drivers of subacute inflammation (peaking at 3–7 days post-ICH) that exacerbate secondary injury through MMP-9-mediated blood–brain barrier disruption (30), whereas NLR primarily reflects acute neutrophil surges and later lymphocyte reconstitution, the latter supporting neural repair in recovery phases (25). This explains SIRI’s superior 1-month prognostic accuracy versus NLR’s relative stability at 6 months. Notably, SIRI>4 specifically flags monocyte-dominated hyperinflammation requiring acute intervention, while NLR > 6 signals sustained adaptive immune dysfunction needing long-term monitoring (31). This provides a novel and valuable reference for clinical staff, enabling them to judge the prognosis of patients effectively and facilitate the formulation of clinical treatment decisions. Hemorrhage has high mortality and disability rates. Secondary injury caused by immunity and inflammation is critical to the rapid progression of deterioration. Without early detection and accurate intervention, it cannot be effectively managed (32, 33). The NLR and SIRI, as effective indicators of the inflammatory response, can accurately reflect the severity and temporal dynamics of inflammation in patients. Inflammatory responses can lead to brain tissue edema and subsequently affect the consciousness of patients, ultimately impacting their prognosis.

Otherwise, there was no interaction between NLR > 6 and SIRI>4, which constitutes one of the significant new findings of this study. These two indicators independently influence the clinical outcomes of patients with ICH stroke. The mechanistic dissociation likely arises from their distinct cellular origins and temporal activation patterns: NLR predominantly reflects acute neutrophil-mediated injury through NETosis and MMP-9 release causing blood–brain barrier disruption, while SIRI captures monocyte/macrophage-driven chronic inflammation via IL-6/TNF-*α* signaling and subsequent fibrotic remodeling (26, 30). This finding implies that clinicians should consider these two indicators separately when evaluating the prognosis of patients. Additionally, further in-depth research is needed to explore the specific mechanisms by which the NLR and SIRI affect the clinical outcomes of ICH stroke patients and to develop more targeted therapeutic strategies.

### Strengths and limitations

This study has several notable strengths. To our knowledge, this is a unique longitudinal investigation examining inflammatory variables in the context of ICH stroke. A particular strength of this research lies in its prospective design and systematic evaluation at three distinct time points. Furthermore, the sample size of this study is another significant advantage. Compared with previous studies, this study included an adequate sample size of participants, which greatly enhanced the statistical ability of our data and the reliability of our conclusions.

While our study has provided valuable insights, it is essential to acknowledge its limitations. First, we did not collect additional information on the medical treatments and complications of these patients after discharge. The use of only the mRS score as the main clinical outcome measure has certain limitations in providing clinical guidance. Additionally, despite our efforts to control for a range of potential confounding variables, there remains the possibility that residual confounding factors were not captured in our analysis. Furthermore, the timing of blood sample collection and the inherent variability of the poststroke period could have introduced some bias into our results. While future studies should monitor how these markers evolve, our baseline numbers already help spot high-risk patients right after admission. Future research, ideally with larger cohorts and standardized blood sampling protocols, will be instrumental in corroborating our findings and elucidating the intricate mechanisms that link inflammatory markers to clinical outcomes in ICH stroke patients.

The scope of future research should be expanded to include larger and longer follow-up multicenter prospective cohorts. This approach will serve not only to validate our findings but also to explore the practical clinical applications of these inflammatory markers, thereby enhancing the evidence base for decision-making in clinical practice. This information will be crucial for advancing our understanding of the role of inflammation in ICH stroke and for developing more targeted therapeutic strategies.

## Conclusion

Our study demonstrates that elevated inflammatory indices (NLR > 6 and SIRI>4) predict poor functional outcomes in ICH patients during post-discharge follow-up, especially at 1-month post-discharge. Notably, SIRI>4 showed superior prognostic value at 3-month follow-up, while NLR > 6 was more predictive at 6-month assessment. These biomarkers may facilitate early risk stratification and personalized treatment planning. Further research should investigate the pathophysiological mechanisms and evaluate whether therapeutic modulation of these indices can improve long-term recovery.

## Data Availability

The raw data supporting the conclusions of this article will be made available by the authors upon reasonable request.
