# Supplementary material for: Neutrophil-to-lymphocyte ratio and systemic inflammation response index as biomarkers for the clinical outcomes of intracerebral hemorrhagic stroke patients: a prospective cohort study
Source: Front Neurol. 2025 Jul 22;16:1616128. doi: 10.3389/fneur.2025.1616128 (PMC12321558; doi:10.3389/fneur.2025.1616128)
Supplement: Supplementary file 1 [file Data_Sheet_1.docx]

**Table S1** The STROCSS guidelines Checklist


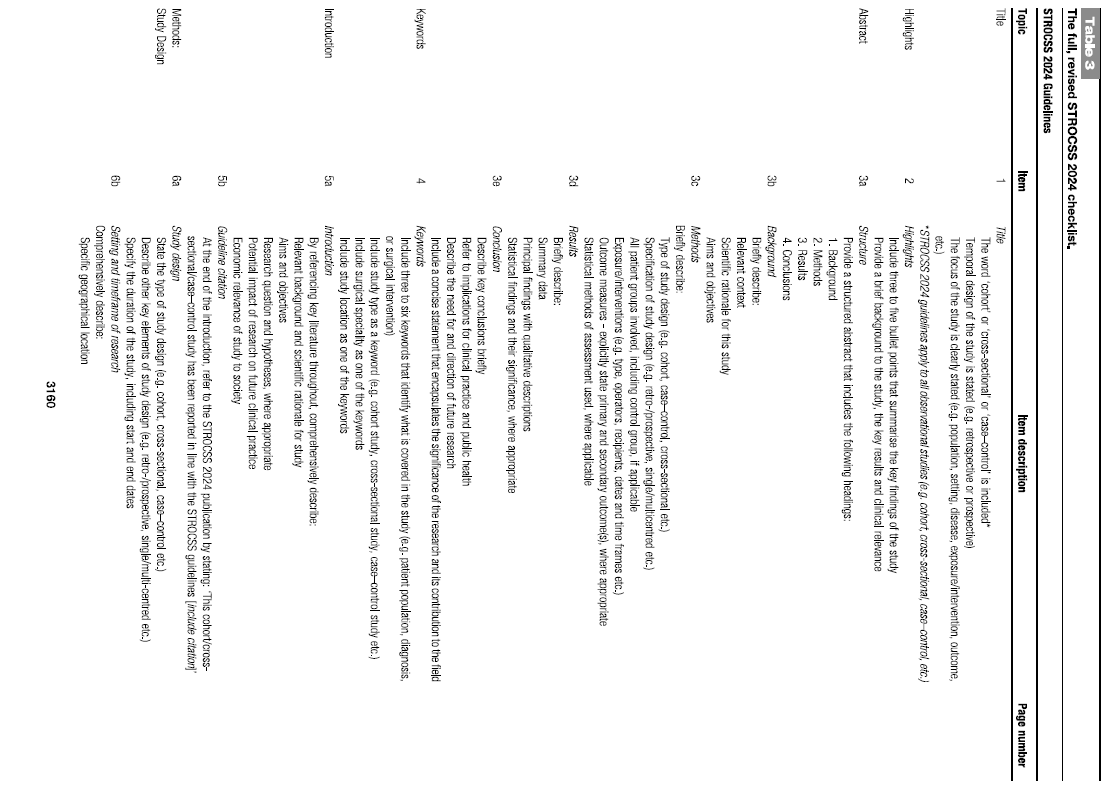


1

1

1

1

1

1

2

2

2

3

3

4


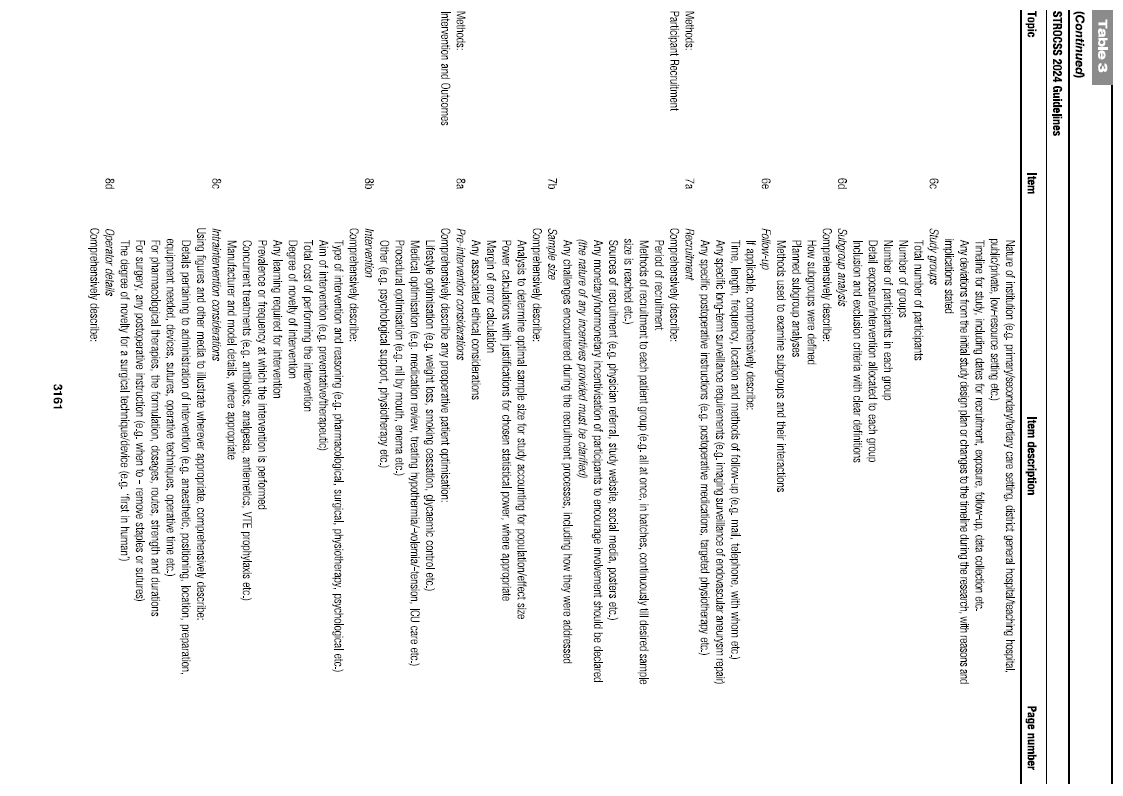


3

3

4

4

4

4

NA


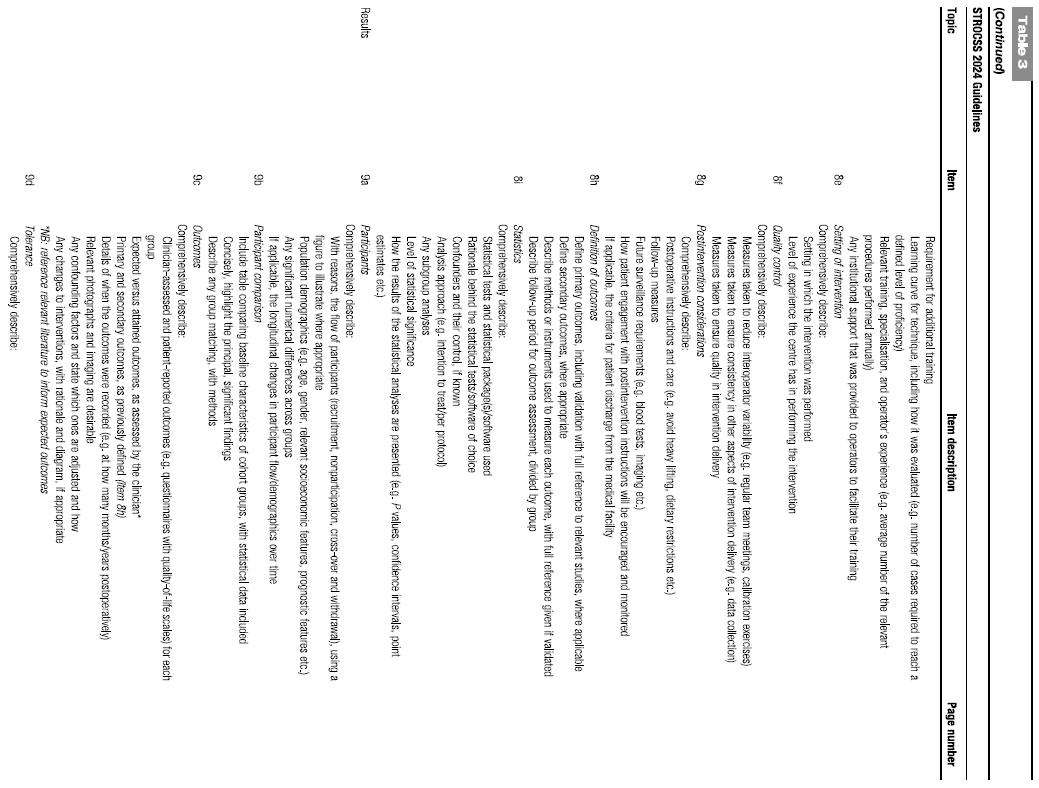


NA

4

4

5

4

5

6


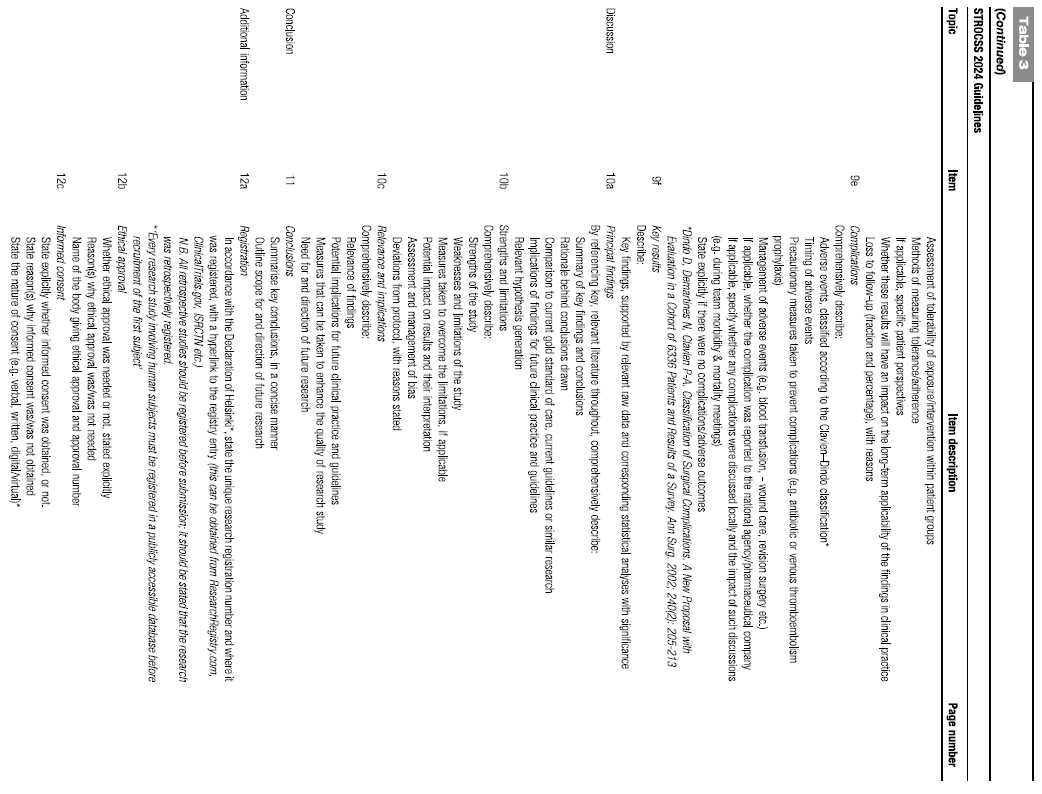


6

6

7

10

11

4

4


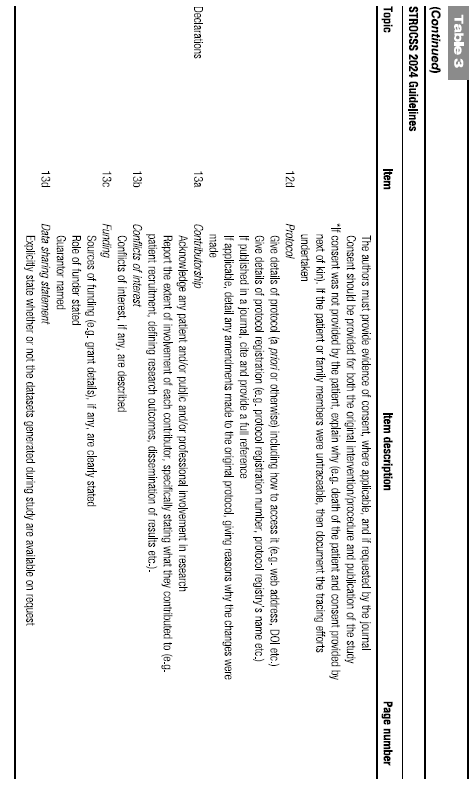


NA

12

12

12

12

**Table S2** Definitions of the severe heart, lung, liver, or kidney disease

| **Diseases** | **Definition** |
| --- | --- |
| severe heart disease | New York Heart Association (NYHA) functional class III or IV; left ventricular ejection fraction (LVEF) < 40%; unstable angina or myocardial infarction within 6 months; severe arrhythmia requiring hospitalization; severe valvular heart disease (e.g., severe aortic/mitral stenosis or regurgitation) |
| severe lung disease | Chronic obstructive pulmonary disease (COPD) requiring long-term oxygen therapy (GOLD grade C or D); interstitial lung disease with resting hypoxemia (SpO₂ < 90% or PaO₂ < 60 mmHg at rest); acute respiratory failure requiring mechanical ventilation (not directly caused by ICH); severe pulmonary hypertension |
| severe liver disease | Child-Pugh class B or C cirrhosis; OR laboratory evidence of significant hepatic dysfunction (serum albumin < 30 g/L AND international normalized ratio (INR) > 1.5 AND total bilirubin > 2× ULN<sup>a</sup>), in the absence of acute reversible causes |
| severe kidney disease | Chronic kidney disease (CKD) stage 4 or 5 (estimated glomerular filtration rate [eGFR] < 30 mL/min/1.73m²); OR requiring maintenance dialysis (hemodialysis or peritoneal dialysis) |

**Table S3** Neutrophil, monocyte and lymphocyte counts at the baseline (n=294)

| **Characteristics** | **1-month post-discharge** | | **3-month post-discharge** ^a^ | | **6-month post-discharge** ^b^ | |
| --- | --- | --- | --- | --- | --- | --- |
|  | **Poor outcome^*^**  **(n=135)** | **Better outcome^*^**  **(n=159)** | **Poor outcome^*^**  **(n=100)** | **Better outcome^*^**  **(n=194)** | **Poor outcome^*^**  **(n=89)** | **Better outcome^*^**  **(n=205)** |
| Neutrophil (10*9/L) | 8.92±3.37 | 7.74±3.19 | 8.82±3.45 | 8.01±3.23 | 8.83±3.47 | 8.04±3.24 |
| Monocyte (10*9/L) | 0.62±0.32 | 0.57±0.33 | 0.60±0.31 | 0.59±0.33 | 0.59±0.31 | 0.60±0.33 |
| Lymphocyte (10*9/L) | 1.02±0.68 | 1.15±0.56 | 1.04±0.75 | 1.12±0.55 | 1.06±0.75 | 1.10±0.56 |
